# Transcriptome of *Pterospermum kingtungense* provides implications on the mechanism underlying its rapid vegetative growth and limestone adaption

**DOI:** 10.1038/s41598-017-03433-1

**Published:** 2017-06-09

**Authors:** Yandong Ren, Yanan Zhu, Qi Wang, Hui Xiang, Boyi Wang

**Affiliations:** 1 State Key Laboratory of Genetic Resources and Evolution, Kunming Institute of Zoology, Chinese Academy of Sciences, Kunming, China. 32 East Jiaochang Road, Kunming, Yunnan Province 650223 China; 2 Yunnan Forestry Technological College. No.1 JinDian, Kunming, Yunnan Province 650224 China; 3 University of Chinese Academy of Sciences, Beijing, 100049 China; 4 Guangzhou Key Laboratory of Insect Development Regulation and Application Research, Institute of Insect Science and Technology & School of Life Sciences, South China Normal University, Guangzhou, 510631 China; 5 Kunming University of Science and Technology, No.727, South Jingming Road, Chenggong District, Kunming, Yunnan Province 650500 China

## Abstract

*Pterospermum kingtungense* C.Y.Wu ex Hsue is a typical tree species living in the relatively adverse limestone habitat. Due to its excellent wood quality and big size, it is an important timber resource which caused its endangered. We firstly provide the data resources by reporting an annotated transcriptome assembly. 203 million unique Illumina RNA-seq reads were produced with totally 50,333 transcripts, among which 48,778 transcripts were annotated. By a global comparison of homology between *P. kingtungense* and cacao, we identified 9,507 single copy orthologues and 990 *P. kingtungense* specific genes. GO enrichment analyses indicate that *P. kingtungense* specific genes are enriched in defense response, implying potential adaptation to limestone environment. As to cell compartment, the genes are enriched in thylakoid component. Consistently, KEGG enrichment indicates that genes are enriched in photosynthesis. In addition, we identified two genes under positive selection in *P. kingtungense* species. These results suggest that *P. kingtungense* have strong photosynthetic capacity, which related to vegetation growth. Our work provides the genomic resources of a limestone specific tree with economic importance to local society and suggests possible mechanism on its characteristics on the limestone adaption and excellent wood properties, which will be important for its conservation and sustainable utilization.

## Introduction

*Pterospermum kingtungense*, belonging to Sterculiaceae, Malvales, is endemic to subtropical evergreen broad-leaved forest on Limestone Mountain of central Yunnan. This species is distributed in the Jingdong County, one of the biodiversity hotspots of CI. The distributions of *P. kingtungense* ranges from 1,400 m to 1,500 m high. Its annual mean temperature is 16.8 °C, with extreme low temperature 10 °C. The community structure exhibits an obvious secondary forest structure and human activities are frequent here. The local soil is typical carbonate soil which has lesser water retention and poor soil fertility conditions^1–3^. Despite, *P. kingtungense* can survive on this kind of soil very well and its wood is superior for its straightness, good hardness and relatively high diameter, which has great economic value in local areas. Besides its excellent wood properties, the bark can be used in traditional Chinese medicine for cure infantile convulsion, waist pain and fracture^4^. Due to its widely application in wooden furniture, the destructive lumbering by local people has been causing the severe decline of the population of *P. kingtungense*. In terms of IUCN criteria, *P. kingtungense* has been listed as critically endangered category^5^.

Recently, scientists have been trying to explore the endangering mechanism of *P. kingtungense*. However and regrettably, researches are still rather few and remained initial and preliminarily descriptive, focusing on survey of the distribution, biological and ecological characteristics of this plant^4, 6, 7^. Nearly no efforts are tried to deepen our understanding on the particular adaption to the limestone soil, excellent wood properties and endangered mechanism of *P. kingtungense* from genetic and genomic view. Currently, with development of sequencing technology and genomics, it is becoming feasible to establish relatively high quality genomic resources for a non-model organism. The *de novo* transcriptome data by RNA-seq is especially useful when genome data is not available. Over the past few years, the next generation sequencing technology has significantly accelerated the speed of gene discovery and is expected to boost genomics studies^8–10^. This technology has been proved to be a valuable addition to evolutionary and ecological research for non-model organisms^11^. Recently, the transcriptome data of many species have been published, such as two invasive whiteflies^12^, *Cusumis melo* ^13^, pennycress^14^ and *Ischnura elegans* ^15^. All these transcriptome data give clues for understanding molecular mechanism of phenotypic adaption of different species and provide important references for further protection and exploitation of the biological resources.

In this study, we for the first time provide the transcriptome of the limestone mountain specific and important local economical tree–*P. kingtungense*. We used the RNA of the seedling to generate the RNA-seq data. By *de novo* assembly and annotation, we obtained the relatively complete geneset information. Then, we compared the geneset of *P. kingtungense* with *Theobroma cacao* which is the most relative species to *P. kingtungense* with genome data available but lives in drastic different habitat from *P. kingtungense*. GO enrichment analysis of *P. kingtungense* specific genes indicates that they are enriched in defense response and thylakoid. KEGG enrichment analysis of *P. kingtungense* specific genes indicated that they are enriched in photosynthesis pathway. We also identified two gene under positive selection in *P. kingtungense*, namely ribosomal protein S4 and NADH dehydrogenase subunit 4. These genes are potential relevant to the limestone specific adaption and rapid vegetative growth, respectively, which helps us to understand the molecular mechanism of limestone specific adaption and rapid vegetative growth of *P. kingtungense*, and provides insights on protection and sustainable application for this valuable natural resources.

## Materials and Methods

### Plant cultivation

The seeds of *P. kingtungense* were collected from Jingdong County (23°56′-24°29′N, 100°22′-101°15′E), Yunnan Province, Southwest China. These seeds were immersed in water for 8 h at 37 °C, and then germinated in agarose culture medium at 25 °C in daylight. After 7–8 days, when the first two leaves of the seedling fully emerged, the whole seedling was harvested and immediately frozen in liquid nitrogen.

### Total RNA extraction

The seedling was grind into powder by pestle and mortal in liquid nitrogen for RNA extraction. Total RNA was extracted using an RNeasy plant mini kit (Qiagen, http://www.qiagen.com) according to the manufacturer’s instructions.

### RNA-seq

To obtain an overview of the *P. kingtungense* transcriptome, polyadenylated RNA was selected using oligo (dT) purification and reverse-transcribed to cDNA and sequenced using the Illumina sequencing platform. The library was size-selected for an insert size of 350 bp and quantified using the Invitrogen PicoGreen dsDNA assay (Life Technologies). The pooled cDNA sample was sequenced using the Illumina HiSeq 2500 platform with 100 bp, paired-end reads.

### Data filter, assembly and annotation

All the raw data were filtered out of the low quality reads, adaptors and removed of the duplication. We also randomly selected 10,000 reads for microorganism contamination detection, and these reads were blasted to the nt database of NCBI using blast software (version 2.2.27, parameter “-p blastn -e 1e-10 -m 8”). Then, all these clean data were assembled into contigs using SOAPdenovo-Trans (version 1.03)^16^ and Trinity (version r2014_01_17)^17, 18^ respectively. As to SOAPdenovo-Trans, K-mers 21, 47, 49, 51, 53, 55, 61, 71 and 81 were tested and other parameters were set as default. As to Trinity, all parameters include K-mers were set as default. Then, we used the results of Trinity because of its better performance. To improve the overall quality of the assembled transcriptome, a two-step quality filtering method was employed^19–21^. Firstly, sequence redundancy was removed by clustering the duplicates using CDHIT-EST (version 4.6.5)^22^ at the cutoff value of 95% sequence similarity and 50% alignment coverage for the shorter sequence. Second, the transcript read coverage at each base was calculated using SOAPaligner (version 2.21)^23^, transcripts that had a mean coverage per base of less than 5 were removed. Then, these contigs were annotated using BLASTX^24^ against different database, including NCBI non-redundant (nr) protein database (ftp://ftp.ncbi.nih.gov/blast/db/nt.Z), InterProScan database^25^, GO database^26^, KEGG database^27–29^, TrEMBL database and SwissProt database. BLASTX were run with e value of 1 × 10^−5^ on all these database.

### Gene expression level

To calculate gene expression, we firstly aligned the reads to the assembled transcriptome, using TopHat (version 2.1.0) with its default parameters. Then, we used Cufflinks to calculate RPKM value of all genes with its default parameters. RPKM value which represents for Reads per Kilobase Million is considered as gene expression level.

### Comparative analyses between *P. kingtungense* and *Theobroma cacao*

We selected *Theobroma cacao*, belonging to the same family with *P. kingtungense* but showing drastic different ecological characters from it. To compare the genesets between these two species, we used blastp to identify homolog genes with the cutoff of 50% identity and e value of 1 × 10^−7^. Those without blast hits are considered as species specific genes. Among these homolog genes, we further identified ortholog genes with reciprocal one-to-one best match. All these specific genes of *P. kingtungense* were performed with GO enrichment and KEGG enrichment analyses. GO enrichment analysis were performed using Bingo in Cytoscape 3.0 package with default parameters^30, 31^. KEGG enrichment was carried out using an R script.

### Molecular evolution

The single copy orthologs between *P. kingtungense* and cacao were used for molecular evolution analysis. To determine whether these genes between *P. kingtungense* and cacao are under selective pressure, we calculated the Ka/Ks values to measure the strength of selection. We aligned coding sequences of the orthologs with codon alignment by MEGA 7^32^ and calculated Ka/Ks of these aligned genes by KaKs_Calculator software^33^.

In order to figure out whether these candidate genes experienced rapid evolution in *P. kingtungense*, the candidate genes with Ka/Ks significantly higher than 1 were further tested selective signal in relatively large evolutionary scale, using PAML 4.8^34^ with the branch model with the CODEML program. We included *Gossypium hirsutum*, *Gossypium harknessii*, *Arabidopsis thaliana*, *Brassica oleracea var. botrytis*, *Brassica napus*, *Raphanus sativus* for the analyses. All the mitochondrion sequences of these species were downloaded from NCBI (GCA_000987745.1, NC_027407.1, KJ820683.1, NC_018551.1, NC_008285.1, and NC_001284.2, respectively) and were further used to reconstruct a species tree. The complete mitochondrial sequences from all 8 plants were aligned using MEGA7, and the aligned sequence is 14,822 base pairs (bp). The dataset of protein coding sequences was subjected to Bayesian analyses in MrBayes (version 3.1.2)^35^. In this analysis, two sets of four simultaneous Markov chain Monte Carlo analyses were run for 1,000,000 generations, with one tree saved every 1,000 generations and the first 25% was discarded as ‘burn-in’. The standard deviation of split frequencies was below 0.01 after 1,000,000 steps, indicating the convergence of the four chains to a stationary distribution. Orthologs of these 8 species were also download from NCBI and codon aligned using MEGA (NC_027406.1, NC_027407.1, NC_001284.2, KJ820683.1, NC_008285.1, NC_018551.1, NC_027407.1, NC_027406.1, NC_003076.8, KJ820683.1, NC_024469.1, NC_008285.1). We test the 1ω model (one-ratio model), 2ω model, 3ω model and 8ω model (all-branches model) respectively. Likelihood ratio was used to test whether the alternative model is better than the null one.

## Results

### RNA-seq data generation and *de novo* assembly of transcirptome

RNA was isolated from whole plant and sequenced on Illumina HiSeq 2500 platform (100 bp paired-end), yielding 179,267,204 reads with a mean quality score >Q30. The full, unfiltered short-read dataset was deposited in the NCBI Short Read Archive (SRA) under the accession number: SRR3621114.

**Figure 1:**
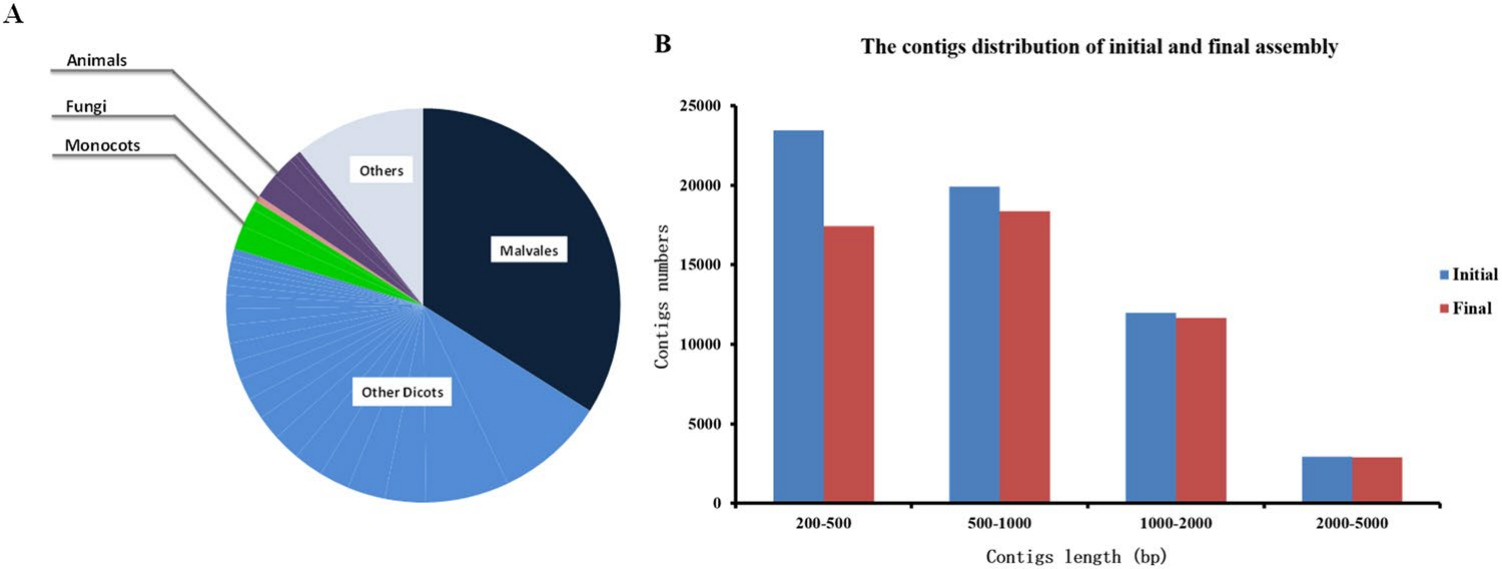
Summary of evaluation on assembly and annotation. (**A**) Top 25 BLASTX hits. Totally, there are six groups were blast hited and individual pieces in each group indicate different families therein. (**B**) Comparison between final (filtered) and initial (un-filtered) transcriptome of *P. kingtungense*. X axis represents transcript length and y represents transcripts frequency, indicating that filtering step proved important for obtaining qualified transcripts as it not only removed of the redundancy but also filtered shorter transcripts.

After removing duplicate reads, trimming adaptors, and filtering for low quality sequences, a total of 44,754,950 unique, clean reads were obtained, with a mean length of 95 bp (Supplemental Table 1). We choose 10,000 reads randomly and blasted to Nr database to detect the microorganism contamination. As shown in Fig. 1(A), top 30 BLASTX orders hits are listed. Of these, Malvales which *P. kingtungense* belongs to, yielded the highest number of top hit (33.967%), followed by Malpighiales and Caryophyllales, and the most species of the top BLAST hits are belong to the Dicotyledons. Most of the reads were blasted to the plants; percentage of top hits to animals is only 3.826%, none of top hits blasted to microorganism. These results indicate that our data are not contaminated by microorganism data.

We used SOAPdenovo-Trans (version 1.03)^16^ and Trinity (version r2014_01_17)^17, 18^ to *de novo* assemble the transcriptome data independently (Table 1). As to the transcriptomes assembled by SOAPdenovo-Trans, The N50 size is from 579 bp to 758 bp with K-mers from 21 to 81. In contrast, the N50 size of the transcriptome assembled by Trinity is 1,135 bp, obviously longer than those by SOAPdenovo-Trans. We therefore choose the transcriptome by trinity for further analyses.

**Table 1:** Assembly results of SOAPdenovo-Trans, Trinity and results after filtering.

Software	K value	Contig N50		Total Length	Total number	Average length	Number > = 1000 bp	Number > = 2000bp	Number > = 5000 bp
		Size (bp)	Number						
SOAPdenovo-trans	21	712	7,547	18,367,311	28,444	645	4,041	622	10
	47	758	7,471	18,474,493	27,420	673	4,397	569	4
	49	750	7,298	17,838,305	26,646	669	4,199	519	5
	51	743	7,110	17,181,676	25,837	665	4,010	469	4
	53	741	6,854	16,453,473	24,871	661	3,824	439	5
	55	733	6,613	15,683,876	23,962	654	3,559	410	4
	61	705	5,672	13,053,199	20,421	639	2,877	302	4
	71	650	3,809	8,059,333	13,314	605	1,579	136	1
	81	579	1,700	3,216,080	5,706	563	506	41	3
Trinity	default	1,135	16,131	58,831,787	67,216	875	19,604	4,237	146
Final assemble results after filtering	none	1,096	12,574	43,720,415	50,333	868	14,571	2,895	74

To improve the overall quality of the assembled transcriptome, a two-step quality filtering method was employed. Firstly, sequence redundancy was removed by clustering the duplicates using CDHIT-EST^22^ at 95% sequence similarity and alignment coverage for the shorter sequence must be more than 50%. This step clustered 13.3% of the transcripts together, leaving 58,253 transcripts. Second, the transcript read coverage at each base was calculated using SOAPaligner^23^. Transcripts that had a mean coverage per base of less than 5 were removed, filtering 7,920 transcripts, and leaving 50,333 high quality transcripts for the following analysis. After the quality filtering, the final transcriptome contained 50,333 high quality transcripts with contig N50 value of 1,096 bp and mean length of 868 bp, which was used for subsequent analyses. From Fig. 1(B), we can find that the more contigs with short length were filtered than contigs with long length. Transcriptome completeness was evaluated by CEGMA (version 2.5)^36^. Totally, 94.92% of 248 ultra-conserved core proteins was identified as ‘complete’ and 97.84% as a ‘partial’ (Supplemental Table 2), consistently indicating high completeness and quality.

### Transcript annotation

Unigenes of the transcriptomes were searched with BlastX against such database as NCBI non-redundant protein database (nr), InterProScan^25^, TrEMBL, SwissProt database, GO^26^, KEGG^27–29^, respectively, with a cut-off of e-value 10-5. About the protein database, the non-redundant protein database (nr) have the highest blast hits, 44,795 (89.0%) transcripts could be matched with NCBI nr database, then 9,610 (19.09%) transcripts can be matched with the TrEMBL database, at last, 5,630 (11.19%) transcripts could be matched with the SwissProt database, 5,032 (10.00%) transcripts could be matched with the InterProScan database. We noticed that the NCBI nr database have the highest match ratio and the other database have a much lower match ration, the reason for this is that nr database is the biggest protein database which means it have the largest number of proteins and many of these proteins were only submitted to the nr database. The assembled transcripts were also annotated with Gene Ontology (GO) into three major GO categories: Biological processes, Cell component and Molecular function. A total of 3,812 (7.57%) transcripts were associated with at least one GO term. Among the Biological processes, “immune system process” and “response to stimulus” may related to the adaption to the reluctant soil and “developmental process” may be concerned to the excellent wood properties of *P. kingtungense* (Supplemental Figure S1). These transcripts were also annotated and classified using Kyoto Encyclopedia of Genes and Genomes (KEGG) database, 4,369 (8.68%) transcripts were assigned to the KEGG database. As to the transcripts, we filtered the cds sequences with unexpected stop codon and shorter than 180 bp and obtained 38,246 cds sequences finally for all subsequent analyses.

### Geneset comparison between *P. kingtungense* and *Theobroma cacao*

We choose cacao (*Theobroma cacao*), the closest species to *P. kingtungense* which genome has been sequenced, for comparison analyses with *P. kingtungense*, in order to probe the possible genset divergence of the two species and further explore the molecular mechanism of the adversity adaptability and growth features characters of *P. kingtungense*. Cacao belongs to the same family with *P. kingtungense*, namely Sterculiaceae. It is well-known that cacao is the source of the world’s cocoa butter and cocoa powder. Cacao is native to humid topics of the central and northern parts of South America. It is commonly cultivated in hot, rainy environments, between 20°N and 20°S of the equator, with maximum cultivation between 10°N and 10°S^37^. Cacao trees are usually planted with fertile soil. Different from the cultivation condition for cacao^4, 37, 38^, *P. kingtungense* lived in such harsh environment as typical carbonate soil which has lesser water retention and poor soil fertility conditions. As to growth and developmental characters, cacao spends a lot of energy on reproductive growth. Usually, cacao seeds are viable for a short time (10–13 weeks) and require up to 50% moisture for germination, while *P. kingtungense*’s blooming period is 4–6 months and blooming period is 7–9 months^4^.

**Figure 2:**
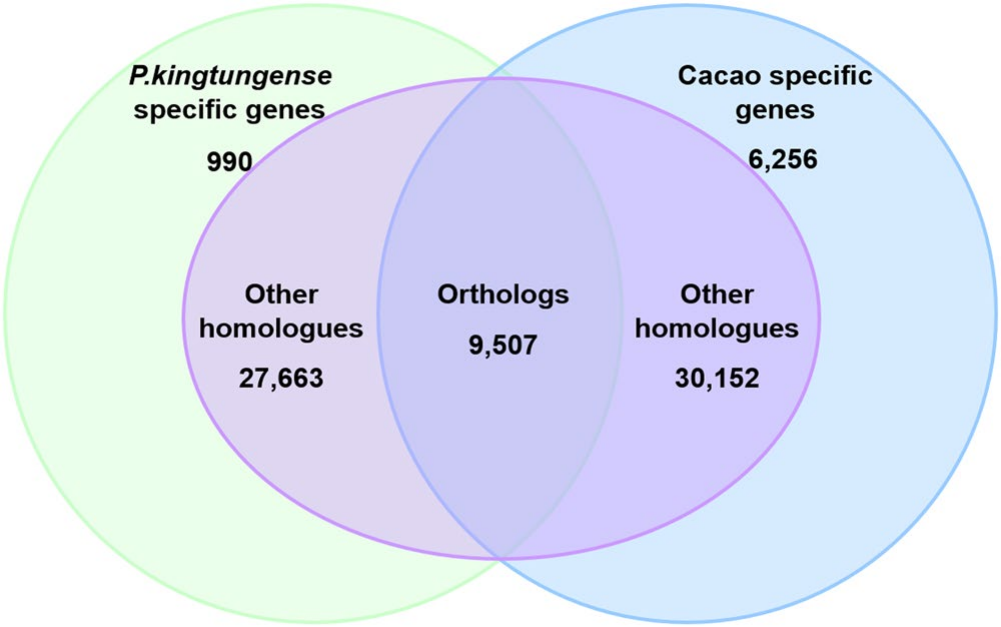
The results of bidirectional blast between *P. kingtungense* and cacao.

Firstly, we identified homologs between *P. kingtungense* and cacao by blastp with a cutoff e value is 10^−7^. About 97.41% of *P. kingtungense* transcripts (37,170 out of 38,160) are homologous to cacao annotated genes and on the contrary, about 86.38% of cacao genes (39,660 out of 45,916) to those of *P. kingtungense*. Furthermore, we identified 9,507 pairs of orthologs by blastp with reciprocal best hit (See Material and methods). Meanwhile, we identified 990 *P. kingtungense* specific and 6,256 cacao specific genes, respectively (Fig. 2).

**Figure 3:**
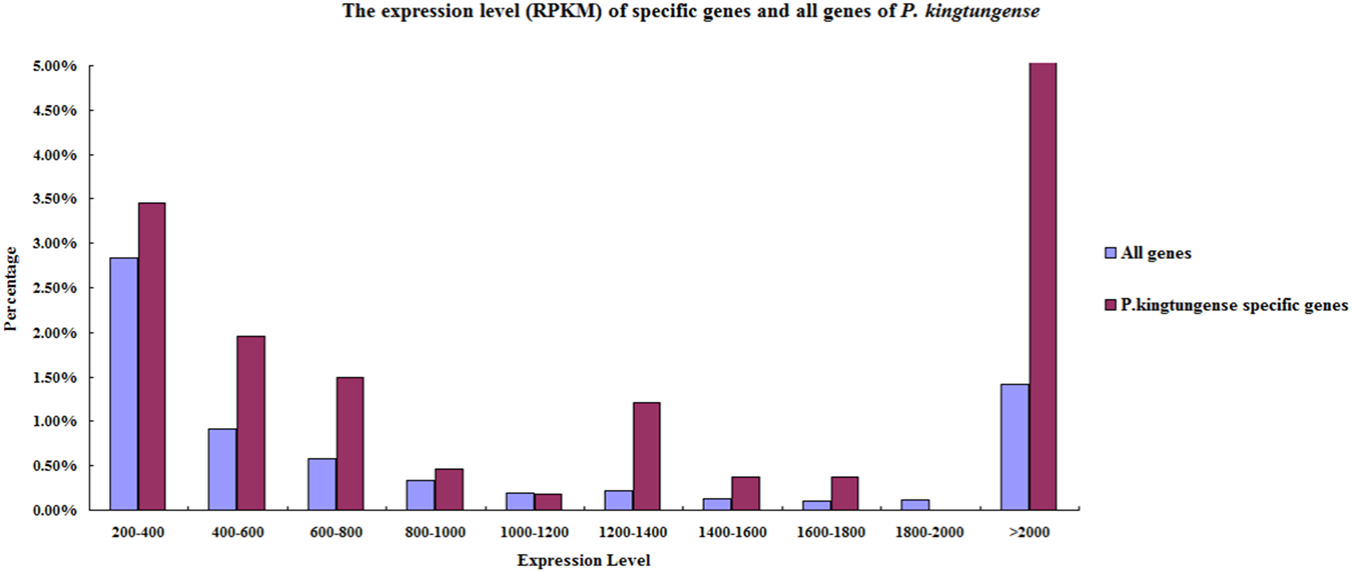
The expression level (RPKM) of specific genes and all genes of *P. kingtungense*.

*P. kingtungense* specific genes are relatively highly expressed among all the transcripts (Fig. 3), suggesting functional importance of them. The GO enrichment analyses of these *P. kingtungense* specific genes showed that they are enriched in the biological process of defense response and the cell compartment “thylakoid”, which is a membrane-bound compartment inside chloroplasts and where the light-dependent reactions of photosynthesis is occurs.

These results suggest that *P. kingtungense* has strong photosynthetic capacity, which may facilitate to its rapid vegetation growth and further contribute to its excellent wood properties^4^. On the other hand, compared to cacao, *P. kingtungense* may need more energy for the adaption of harsh environments (Table 2). KEGG enrichment analysis indicated that the genes are enriched in photosynthesis and oxidative phosphorylation (Table 2). These results are consistent to the GO enrichment results and indicate that besides the strong photosynthetic capacity *P. kingtungense* also have produced massive of energy, which might be associated with fast growth rate and response process for the adaption of harsh environments.

**Table 2:** Functional character of *P. kingtungense* specific genes.

GO-ID / KEGG Map Number	GO Category	Description	p-value	Gene List
GO-ID: 6952	Biological Process	Defense response, physiological defense response, antimicrobial peptide activity, defense/immunity protein activity	1.3554E-3	*P*. *kingtungense*_15448, *P*. *kingtungense*_28133, *P*. *kingtungense*_28134, *P*. *kingtungense*_30825, *P*. *kingtungense*_28135
GO-ID: 9579	Cellular Component	Thylakoid	7.6134E-3	*P*. *kingtungense*_28393, *P*. *kingtungense*_15680, *P*. *kingtungense*_15679
GO-ID: 44436	Cellular Component	Thylakoid part	7.6134E-3	*P*. *kingtungense*_28393, *P*. *kingtungense*_15680, *P*. *kingtungense*_15679
Map: 00195	—	Photosynthesis	1.5876E-2	*P*. *kingtungense*_28393, *P*. *kingtungense*_15679, *P*. *kingtungense*_15680
Map: 00190	—	Oxidative phosphorylation	3.8184E-2	*P*. *kingtungense*_13423, *P*. *kingtungense*_837, *P*. *kingtungense*_890, *P*. *kingtungense*_18131

### Analysis of molecular evolution

We test the selective signal of the 9,507 orthologs between *P. kingtungense* and cacao by Ka/Ks ratio and detected 2 orthologous gene pairs under strong selective signal (Ka/Ks ratio larger than 1 and p < 0.01). One is ribosomal protein S4 and the other is NADH dehydrogenase subunit 4. Plant ribosomal protein S4 has long been implicated in diverse functional activities. It is an antitermination transcription factor^39^. In maize, it also functions as a general endoprotease capable of abrogating protein synthesis^40^. We therefore suspect that selection on this highly expressed of ribosome protein S4 thus might associated with biosynthesis for rapid vegetation growth in *P. kingtungense*. NADH dehydrogenase subunit 4 of mitochondrion, which related to the ATP synthesis, coupled electron transport which will meet the energy requirements for *P. kingtungense*.

We further used PAML and branch model therein to test whether these genes are rapid evolved in *P. kingtungense*. We test different models as follows (Table 3); 1ω model (one-ratio model), 2ω model, 3ω model and 8ω model (free branches model), respectively.

**Table 3:** Results of Tests of the Differences in dN/dS Ratios between *P. kingtungense* and others.

Gene name	Model	Categories	INL	P value (LRT)
NAHD	1ω model	—	−2671.893	—
	2ω model	1. Sterculiaceae/others	−2686.736	5.085e-08 vs 1ω
	2ω model	2. *P. kingtungense*/others	−2670.300	7.418e-02 vs 1ω
	3ω model	1. *P. kingtungense*/Sterculiaceae/others	−2684.116	7.646e-07 vs 1ω
	3ω model	2. Cacao/Sterculiaceae/others	−2686.089	9.908e-08 vs 1ω
	8ω model	All branches	−2666.445	9.742e-04 vs 1ω
Pros4	1ω model	—	−4828.380	—
	2ω model	1. Sterculiaceae/others	−4818.044	5.451e-06 vs 1ω
	2ω model	2. *P. kingtungense*/others	−4823.429	1.649e-03 vs 1ω
	3ω model	1. *P. kingtungense*/Sterculiaceae/others	−4814.722	1.728e-07 vs 1ω
	3ω model	2. Cacao/Sterculiaceae/others	−4815.089	2.527e-07 vs 1ω
	8ω model	All branches	−4799.172	2.121e-14 vs 1ω

As to gene ribosomal protein S4, we firstly used the one-ratio model, a very strict model that allows only a single Ka/Ks ratio for all branches. The ω value is 0.47206, providing good support for the expected presence of negative selection on it, indicating that strong purifying selection plays a central role in the evolution of mtDNA to keep its important functions in energy metabolism^41–45^. Then, we test the 8ω model (free branches model) versus the 1ω model indicated that the free ratio model is better than the 1ω model (p = 2.121e-14 ≪ 0.01, likelihood ratio test), suggesting that there are different evolutionary rate in different branch. We further tested the 2ω2 model (*P. kingtungense*/others vs the 1ω model) and also found it better than the 1ω model. The *P. kingtungense* branch had a significantly higher ω value than the other branches (468.5347 and 0.3978, respectively, P = 0.074, likelihood ratio test), suggesting that ribosomal protein S4 fast evolved in *P. kingtungense*. In another 2ω1 model (Sterculiaceae/others), the Sterculiaceae branch had a significantly lower ω value than the other branches (0.0375 and 0.6613, respectively, P = 5.085e-08, likelihood ratio test), suggesting that rapid evolution didn’t occur in Sterculiaceae. In the 3ω1 model (*P. kingtungense*/Sterculiaceae/others), the Sterculiaceae branch had a significantly lower ω value than the other branches (0.0164 and 0.5458, respectively) and the *P. kingtungense* branch had a significantly higher ω value than the other branches (2.1918 and 0.5458, respectively, P = 7.646e-07, likelihood ratio test). Based on the above results, we can infer that fast evolution occurred specifically at *P. kingtungense* branch.

Similarly, as to NAHD, we used the one-ratio model and the ω value for all 8 individual genes is 0.91831, implying that overall this gene might be not under strong functional constraint. However, free ratio model, which is better than one ratio model (p = 9.742e-04, likelihood ratio test), indicates that there are drastic different ω values among different branches, providing a possibility that this gene might be under selection in certain branches. So we test different branches to catch the model with different ratio. At first, we used 2ω1 model (Sterculiaceae/others) and found that Sterculiaceae branch had a significantly lower ω value than the other branches (0.4941 and 0.9209, respectively, likelihood ratio test [LRT] test: P = 5.085e-08, suggesting that this gene might subject to functional constraint in Sterculiaceae branch. Further, we used the 3ω1 model (*P. kingtungense*/Sterculiaceae/others to test whether the evolution of this gene in *P. kingtungense*. The Sterculiaceae branch still had a significantly lower ω value (0.0001) than the other branches 0.8254, but the *P. kingtungense* branch had a significantly higher ω value than the other branches (p = 7.646e-07). In summary, we can conclude that the Sterculiaceae branch do have a fast evolutionary rate. However, the *P. kingtungense* branch may have a fast evolutionary rate due to its much higher ω value than the other branches and the P value (P = 7.418e-02) is a marginal significant. In the 3ω1 model (*P. kingtungense*/Sterculiaceae/others), the *P. kingtungense* branch may have a fast evolutionary rate due to its much higher ω value (999) than the other branches (0.8254) and the P value is less than 0.05. So we can infer that the Sterculiaceae branch do not have a fast evolutionary rate and the *P. kingtungense* branch may have a fast evolutionary rate. As mentioned before, *P. kingtungense* have excellent wood properties due to its strong photosynthetic capacity and two rapid evoluted genes, the ability of vegetative growth is much better than reproductive growth, which impede the reproduce of it and may cause the endangered of it.

These two genes may play important roles in vegetation growth of wood. As mentioned above, ribosome protein S4 might repress protein might favor on its ability on abrogating protein synthesis and thus contribute to vegetation growth. Meantime, NAHD provides energy for this process. These results might to some extent, explain the straightness, big bore and hardness of the *P. kingtungense* woods^4^.

### Summary

In this study, we for the first time provide transcriptome data resources of a limestone specific endanger and important economic tree, *P. kingtungense*. These data are expected to provide comprehensive information relevant to the effective conservation and sustainable utilization of resources.

*P. kingtungense* lived in the limestone and this kind of soil is quite special. Limestone is lack of water and nutrient. So the plants living in the limestone must have strong defense response to water stress, poor soil stress and some biotic stimulus. Besides these, according to the previous research, limestone specific plants also have an efficient utilization of sun-light^46^. From the functional analysis of *P. kingtungense* specific genes, both GO enrichment and KEGG enrichment show that these specific genes were about photosynthesis, and GO enrichment analysis also show that *P. kingtungense* have strong defense response.

*P. kingtungense* is an important economic local tree and popular for its excellent wood properties. So its wood is widely used for house construction and unfortunately this is one of the most important reasons that cause it endangered. It is well known that the growth of wood depends on the vegetable growth of trees, which helped *P. kingtungense* adapt to the poor soil by efficient use of sun light. Our enrichment of *P. kingtungense* specific genes analysis shows that *P. kingtungense* have strong photosynthesizing capacity which explained the excellent wood features. Besides, these two rapid evolution genes were all about the protein synthesis. These results also hinted about the reasons of its excellent wood properties.

In summary, we have started a new beginning of the *P. kingtungense* research, especially in the adaption of the environmental stress. Our analysis provides important insights into the lack of water stress, poor soil stress and some biotic stimulus. Importantly, we found that *P. kingtungense* is quite different from cacao in its vegetable growth and response to stress adaption. We suspect that *P. kingtungense* have strong adaption to the limestone soil and spend too much energy for the vegetable growth and stress adaption. In addition, due to its excellent wood properties, it’s over-cutting by local people. All of this caused it to become endangered. Next-generation sequencing technologies, such as genome-seq, may be helpful for our future studies, and with the development of genome editing technique, all these valuable genes can be applied in other species, which will help to sustainable utilization of the genetic resources of *P. kingtungense*.

## Electronic supplementary material

Supplementary information
