# Supplementary material for: Transcriptome of Pterospermum kingtungense provides implications on the mechanism underlying its rapid vegetative growth and limestone adaption
Source: Sci Rep. 2017 Jun 9;7:3198. doi: 10.1038/s41598-017-03433-1 (PMC5466617; doi:10.1038/s41598-017-03433-1)
Supplement: Supplementary file 1 — Supplementary information [file 41598_2017_3433_MOESM1_ESM.doc]

**Transcriptome of *Pterospermum kingtungense* provides implications on the mechanism underlying its rapid vegetative growth and limestone adaption**

Yandong Ren^a, b, c, 1^, Yanan Zhu^e, a, 1^, Qi Wang^b^, Hui Xiang^d,a,*^ Boyi Wang^b,1,*^,

a. State Key Laboratory of Genetic Resources and Evolution, Kunming Institute of Zoology, Chinese Academy of Sciences, Kunming, China. 32 East Jiaochang Road, Kunming, Yunnan Province, China, 650223

b. Yunnan Forestry Technological College. No.1 JinDian, Kunming, Yunnan Province, China, 650224

c. University of Chinese Academy of Sciences, Beijing, China, 100009

d. Guangzhou Key Laboratory of Insect Development Regulation and Application Research, Institute of Insect Science and Technology & School of Life Sciences, South China Normal University, Guangzhou, 510631, China

e. Kunming University of Science and Technology. No.727, South Jingming Road, Chenggong District, Kunming, Yunnan Province, China, 650500

**1. These authors are equally contributed.**

*** For correspondence: Boyi Wang: wangboyi1978@163.com; Hui Xiang: xiang_shine@foxmail.com**

**
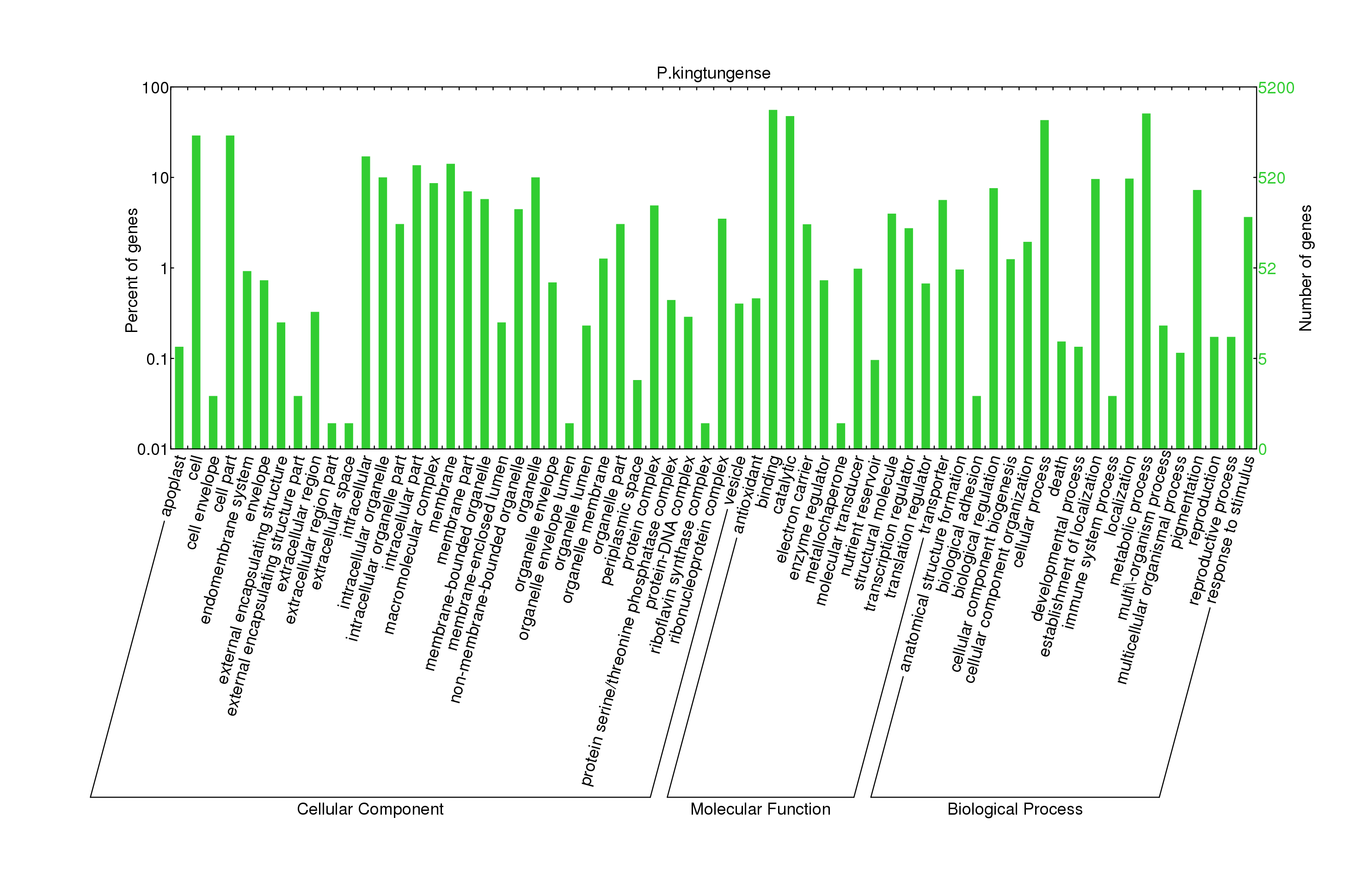
**

**FIG.S1 The GO terms for the transcriptomic sequences of *P. kingtungense*.**

**Table 1.** Statistics of raw data and clean data after filtering.

| **Reads Type** | **Library ID** | **Insert Size (bp)** | **Reads Length (bp)** | **Reads Number (M)** | **Bases Number (M)** |
| --- | --- | --- | --- | --- | --- |
| Raw reads | BWY2 | 350 | 100 | 179.267204 | 17926.7204 |
| Clean reads | BWY2 | 350 | 96 | 44.754950 | 4296.4752 |

**Table 2 CEGMA test results**

| **Category** | **#Prots** | **%Completeness** |  | **#Total** | **Average** | **%Ortho** |
| --- | --- | --- | --- | --- | --- | --- |
| **Complete** | 197 | 79.44 |  | 721 | 3.66 | 94.92 |
| **Group 1** | 46 | 69.70 |  | 156 | 3.39 | 91.30 |
| **Group 2** | 42 | 75.00 |  | 154 | 3.67 | 94.48 |
| **Group 3** | 48 | 78.69 |  | 189 | 3.94 | 100.00 |
| **Group 4** | 61 | 93.85 |  | 222 | 3.64 | 96.72 |
|  | | | | | | |
| **Partial** | 231 | 93.15 |  | 939 | 4.06 | 97.84 |
| **Group 1** | 58 | 87.88 |  | 231 | 3.98 | 98.28 |
| **Group 2** | 51 | 91.07 |  | 210 | 4.12 | 96.08 |
| **Group 3** | 57 | 93.44 |  | 237 | 4.16 | 98.25 |
| **Group 4** | 65 | 100.00 |  | 261 | 4.02 | 98.46 |
